# Morphogenetic identification of a new record *Deudorix livia* (Lepidoptera: Lycaenidae) in Assiut Governorate of Northern Upper Egypt

**DOI:** 10.1038/s41598-023-46231-8

**Published:** 2023-11-16

**Authors:** Farouk A. Abdel-Galil, Sara E. Mousa, Gaber H. Abou-Elhagag, Ahmed M. M. Ahmed, Ammar Al-Farga, Mohammad Allam, Mervat A. B. Mahmoud

**Affiliations:** 1 Plant Protection Department, Faculty of Agriculture, Assiut University, Assiut, 71526 Egypt; 2 Department of Biochemistry, College of Sciences, University of Jeddah, 21959 Jeddah, Saudi Arabia; 3 Department of Zoology, Faculty of Science, Luxor University, Luxor, Egypt; 4 Zoology Department, Faculty of Science, South Valley University, Qena, Egypt

**Keywords:** Zoology, Entomology

## Abstract

*Deudorix livia* (Klug, 1834) (Lepidoptera: Lycaenidae) is one of the most serious lepidopteran insect pests attacking pomegranate fruit around the world, including Egypt (Assiut Governorate, Upper Egypt). To create an effective program (IPM) to control such harmful pests, accurate identification of the pest morphology and genetic structure is essential. Studies on the morphogenetics of this pest are scarce. So, the goal of this research is to identify it both morphologically and genetically. Pomegranate butterfly immature stages were collected from infested pomegranate fruits and reared in the laboratory until the adult's emergence. By using light and scanning electron microscopy, some morphological structures of males and females were studied. DNA was extracted from the legs of a pomegranate butterfly adult. Also, PCR was conducted by using the mitochondrial *CO1* gene for sequencing and phylogenetic tests. The results show that the body scales are a mixture of dark and light gray on the dorsal side and white on the ventral side in both sexes. The average male body length (BL) was 11.674 ± 0.299 mm and was 11.458 ± 1.001 mm for the females. The wing venation is similar in both sexes. For the first time, a partial sequence of the mitochondrial *CO1* gene in *D. livia* was deposited in GenBank (MW463927).

## Introduction

*Punica granatum* L. is one of the most important worldwide crop fruits. Pomegranate fruits possess medical and nutritional values for humans due to their contents of antioxidants, vitamins, potassium, calcium, magnesium, iron, and zinc^1^.

Unfortunately, fruits are infested with a wide range of insect pests. The most serious pests are lepidopteran insects such as pomegranate butterfly *Deudorix* (= *Virachola*) sp., (Lepidoptera: Lycaenidae) (Klug), fruit sucking moth *Eudocima* sp., (Clerk) (Lepidoptera: Erebidae), carob moth *Ectomyelois* (= *Apomyelois* and *Spectrobates*) sp., (Zeller) (Lepidoptera: Pyralidae), and honeydew moth *Cryptoblabes* sp. (Lepidoptera: Pyralidae)^2^.

In Egypt, the pomegranate butterfly is one of the most dangerous insect pests infesting the pomegranate fruits and the most spread. So, the first step for pest control and designing an appropriate Integrated Pest Management (IPM) program is to begin with accurate identification of the target pest. The real information about the pomegranate butterfly is still recorded from non-taxonomical resources. Even though many authors have identified lepidopteran species morphologically, the morphological criteria are not accurate enough to differentiate among these species. Additionally, some small invisible changes in morphological characteristics may remain unnoticed^3^ and not enough for their identification and must be supported by molecular identification methods.

In this regard, the molecular approach is an efficient support for species identification, especially when there are many ambiguities in conventional tools^4,5^**.** The mitochondrial DNA sequences are important tools for species-level identification. Especially, the mitochondrial genes and the cytochrome oxidase subunit 1 (*CO1*) which are used as standard barcode regions for several insect species^6^**.** Using *CO1* has proved to be very useful not only for identifying species but also for revealing cryptic species^7^. So, the objectives of the current study are to accurately identify the pomegranate butterfly type infesting pomegranate orchids in Assiut Governorate, Northern Upper Egypt, which produces 27.93% of the world's pomegranate crop^8^ by morphological descriptions and genetic techniques.

## Materials and methods

### Experimental area

This study was conducted in Experimental Farm, Faculty of Agriculture, Assiut University, Assiut, Northern Upper Egypt (375 km South of Cairo) for collecting different stages of pomegranate butterfly from pomegranate fruits.

### Pomegranate butterfly collection

**Figure 1:**
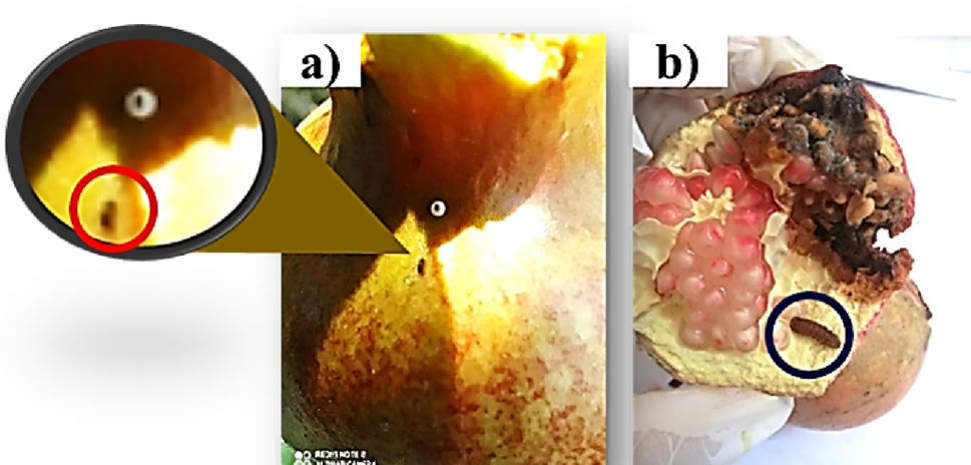
Immature stages of pomegranate butterfly (eggs and larvae) collected from infested fruits; (**a**) a red circle surrounds 1st larval instar hatched from the egg, and (**b**) a black circle surrounds the 4th larval instar with symptoms of infestation caused by larvae that inter-feeding fruits.

The immature stages (eggs, larvae, and pupae) were collected from infested fruits **(**Fig. 1**)** of pomegranate, *Punica granatum* L. (Myrtales: Punicaceae). The samples were incubated in The Biological Control Lab., Plant Protection. Dept., Assiut Univ., Assiut, Egypt, at (27 ± 1 °C, 70 ± 10% RH, and L 16: D 8).

### Morphological identification

Body coloration, wing venation, leg structure, and body morphometrics are the morphological criteria used for identifying pomegranate butterfly^9,10^**.**

#### Scanning electron microscopy (SEM)

Skeletal structures were studied using a Jeol JSM-5500. LV(JEOL-Japan) scanning electron microscope in The Central Lab., Fac. of Sci., South Valley Univ.

#### Light microscope

Wing scales were removed according to Belkin^11^, and the bleached wings were mounted on slides for studying wing venation by using the Leica DC150 Camera.

#### Body morphometrics

**Figure 2:**
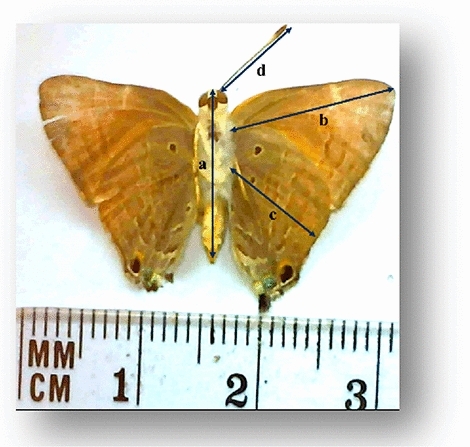
The measured morphometric criteria of pomegranate butterfly “ventral view”: (**a**) body length (BL), (**b**) forewing length (FWL), (**c**) hindwing length (HWL), and (**d**) antennal length (AntL).

The criteria of measurements were body length (BL = from the head to the tip of the abdomen (Fig. 2a)), fore wing length (FWL = from the base (humeral angle) to the apex (apical angle) (Fig. 2b)), hind wing length (HWL = from the base to the middle of the term (apical margin), (Fig. 2c)), and antennal length (AntL = from the base to the tip of the antenna (Fig. 2d))^10^. Measurements determined by the HDMI MULTI-OUTPUT HD’ (Toup Cam_120) CAMERA.

### Molecular genetic identification of pomegranate butterfly

#### DNA isolation

DNA was extracted from the specimen’s legs from a pomegranate butterfly and followed by a modified cetyltrimethylammonium bromide protocol with an additional polyethylene glycol precipitation as described by Reineke^12^.

#### Polymerase chain reaction (PCR) conditions

The mitochondrial *CO1* gene was amplified using the primers LepF1 (ATTCAACCAATCATAAAGATATTGG) and LepR1 (TAAACTTCTGGATGTCC-AAAAAATCA)^7^. The PCR reactions comprised 1μL (10 pmol) of each forward and reverse primer, 1μL of genomic DNA, and 20μL PCR master mix in a final reaction volume of 40μL. The PCR was carried out under the following conditions: denaturing at 95 °C for 4 min., followed by 34cycles of denaturing at 94 °C for 60 s., alignment at 48 °C for 60 s., and extension at 72 °C for 60 s., finishing with an extension at 72 °C for 7 min. All PCR products were visualized using 1.5% agarose gel stained with ethidium bromide. Gel electrophoresis was run for 40 min., at 100 V using 100 bp DNA Ladder RTU (Ready-to-Use) GeneDireX.

#### Sequence and phylogenetic analysis

All DNA sequencing was achieved by Macrogen (Seoul, South Korea). The sequences were deposited at the National Centre for Biotechnology Information (GenBank/NCBI) to obtain the accession number. DNA sequences were primarily aligned with the default parameters of CLUSTALW^13^. Two methodologies, Minimum Evolution (ME) and Neighbour-joining (NJ) implemented in MEGA software version 7.0 18^14^, were used for phylogenetic reconstructions. In this study, 1000 bootstrap iterations^15^ were applied. Sequence divergences were calculated using Kimura 2-parameter distances^16^.

#### GenBank accession number

The partial sequence of the *CO1* region of the butterfly reported in this paper is deposited in GenBank nucleotide sequence databases (http://www.ncbi.nlm.nih.gov) under accession number MW463927 for DAUF-Pomegranate.

### Data analysis

Means ± standard deviation (SD) was determined by using Microsoft Excel 2016 for morphometric measurement data.

## Results

### Morphological identification

Using light microscopy and scanning electron microscopy (SEM), some morphological structures of male and female pomegranate butterfly were illustrated.

#### The general body coloration

**Figure 3:**
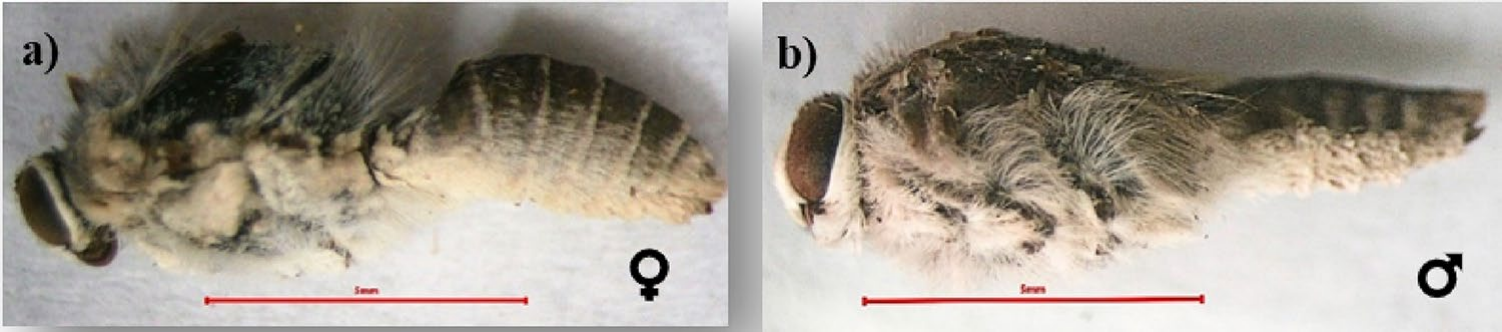
The body scales color of pomegranate butterfly adults: (**a**) female and (**b**) male.

The general color of body scales is a mix of dark and light grey on the dorsal side and white on the ventral side in both sexes **(**Fig. 3**)**.

#### The head

##### Compound eyes

**Figure 4:**
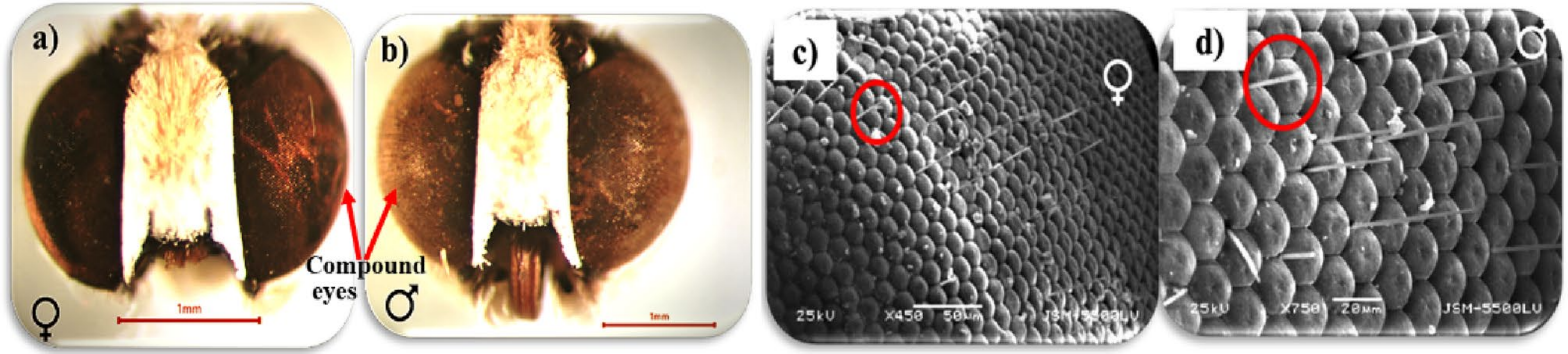
The head capsule of a pomegranate butterfly: (**a**,**b**) Light micrograph, (**c**,**d**) SEM, red circle surrounds the interfacial hair in a corner between three facets.

The color of compound eyes was brown and coppery, (Fig. 4a,b). For the first time study by focusing on one scanned eye, it there are the interfacial hairs (Fig. 4c,d) were found distributed across the eye in a corner between three facets.

##### Antennae

**Figure 5:**
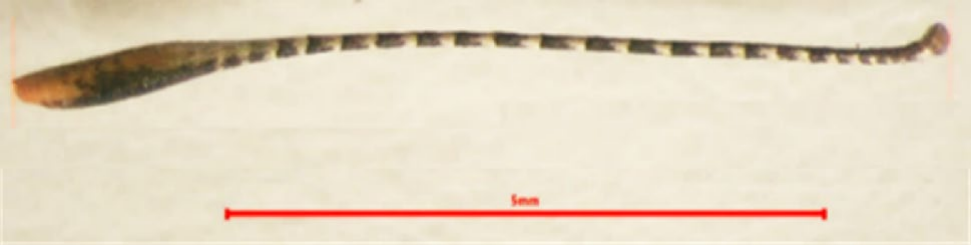
Clavate type of pomegranate butterfly antenna by using light microscopy.

The antenna of the pomegranate butterfly is clavate type **(**Fig. 5**)**. The number of segments in the flagellum is 22 filiform segments followed by 17 clubbed ones in both sexes. The color of terminal segments is orange and black, but other segments are black and white.

##### Mouthparts

**Figure 6:**
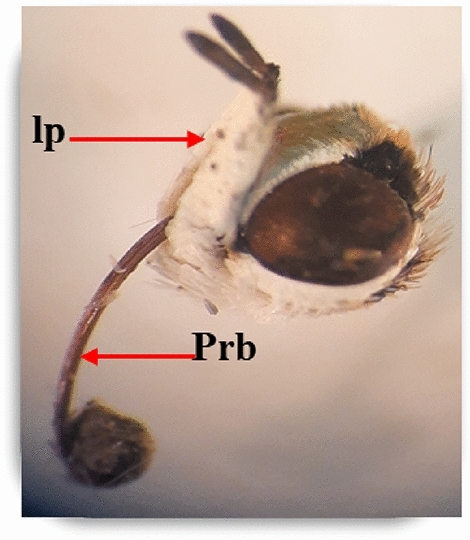
Mouthparts of pomegranate butterfly: light micrograph head capsule (x = 16): Lp: labial palp and Prb: proboscis.

Mouthparts of pomegranate butterfly are sucking type. There are a pair of three-segmented labial palps and a brown coiled proboscis (galea). The labial palps tip is covered with brown scales and the bases with white scales **(**Fig. 6**)**.

#### The thorax

In both sexes, the coloration of the thorax is shiny dark grey, and the smallest of the three thoracic segments is the prothorax in a pomegranate butterfly.

#### Legs

**Figure 7:**
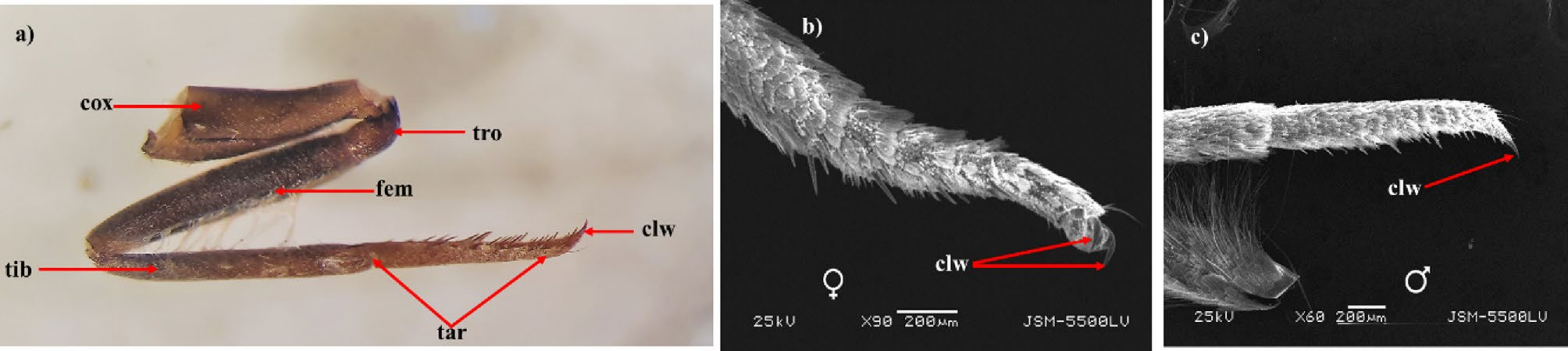
The Legs of pomegranate butterfly: (**a**) light micrograph of the leg without scales (x = 16), (**b**) SEM of the male foreleg, and (**c**) SEM of female foreleg: cox: coxa, tro: trochanter, fem: femur, tib: tibia, tar: tarsus, and clw: claw.

The legs of a pomegranate butterfly are walking type. The three pairs of legs are covered with white scales. Each leg consists of a coxa, trochanter, femur, tibia, and five segmented fused tarsi **(**Fig. 7a**)**. Tibiae of legs apically bear well-developed branched spurs. The apical tarsomere bears two claws with pulvilli and empodium in all legs for both sexes except the foreleg of the male consists of one claw **(**Fig. 7b,c).

#### Wings

##### Wing coloration

Both sexes of pomegranate butterfly differed in their coloration.

##### Upper side of female and male wing

**Figure 8:**
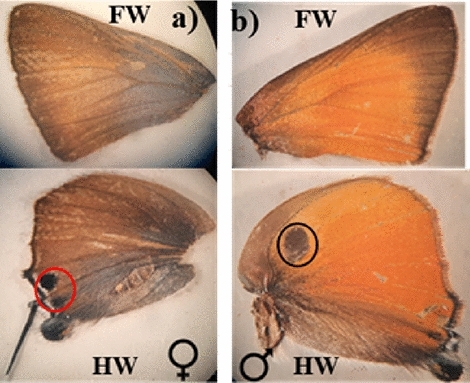
The wing upper side of a pomegranate butterfly (x = 8): (**a**) The female wing's upper side (a red circle surrounds two spots) and (**b**) The male wing's upper side (a black circle surrounds one spot), FW: fore wing and HW: hind wing.

The female fore wing upper side scaled dark brown in the apical angle (apical and sub-apical area) and apical margin (marginal and sub-marginal area), brown color in the post-discal and discal area, and a bluish tint becoming darker in the humeral angle (basal and post-basal area) (Fig. 8a FW). The female hind wing's upper side is covered in brown scales except for the anal margin (tornal area) and humeral angle (part of the discal, sub-discal, basal, and dorsal areas), which are squirrel color. Also, two spotted patches were recognized nearby the anal angle (tornal area), (Fig. 8a HW).

The upper side of the male forewing is covered by dark orang scales, except for the costal margin (costal area), apical angle (apical and subapical area), and apical margin (marginal area), which are brown. Except for the humeral angle (basal area), anal margin (dorsal area), and part of (sub-discal, discal, and post-discal area) (Fig. 8b FW). The male hind wing's upper side is covered by dark orange scales. Additionally, there was one spotted patch in the sub-discal area and near the humeral angle (basal area) (Fig. 8b HW).

##### Lower side of female and male wing

**Figure 9:**
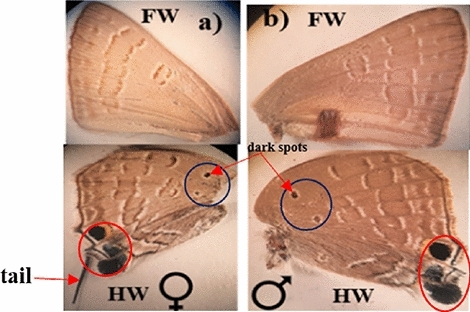
Wing coloration of the lower side of pomegranate butterfly (x = 8): (**a**) The female wing's lower side, (**b**) The male wing's lower side (a red circle surrounds two spots female and male), FW: Fore Wing and HW: hind wing.

The lower sides of the wings are similar in both sexes (Fig. 9). The fore and hind wings’ lower sides are covered with grey scales with different scattered bands. There are two black rounded spots, one just above the filamentous tail and the other on the wing tip. It is worth pointing out herein that in the sub-discal area of the hindwing lower side, clearly encounter two dark spots surrounded by a white oval circle (Fig. 9a,b HW).

##### Wing venation

**Figure 10:**
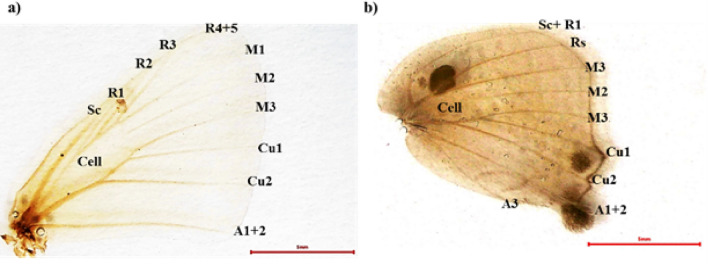
Wing venation of pomegranate butterfly: (**a**) forewing veins, (**b**) hindwing veins (Sc: subcostal, R: Radius, M: Media, Cu: Cubitus, and AL: Anal).

The venation in pomegranate butterfly is similar in both sexes as described in the family individuals by^17^. The forewing with 11 veins: Sc, R1, R2, R3, R4 + 5, M1, M2, M3, Cu1, Cu2 and A1 + 2, (Fig. 10a). The hind wing with 9 veins, include Sc + R1, Rs, M1, M2, M3, Cu1, Cu2, A1 + 2 and A3, (Fig. 10b).

#### The abdomen

**Figure 11:**
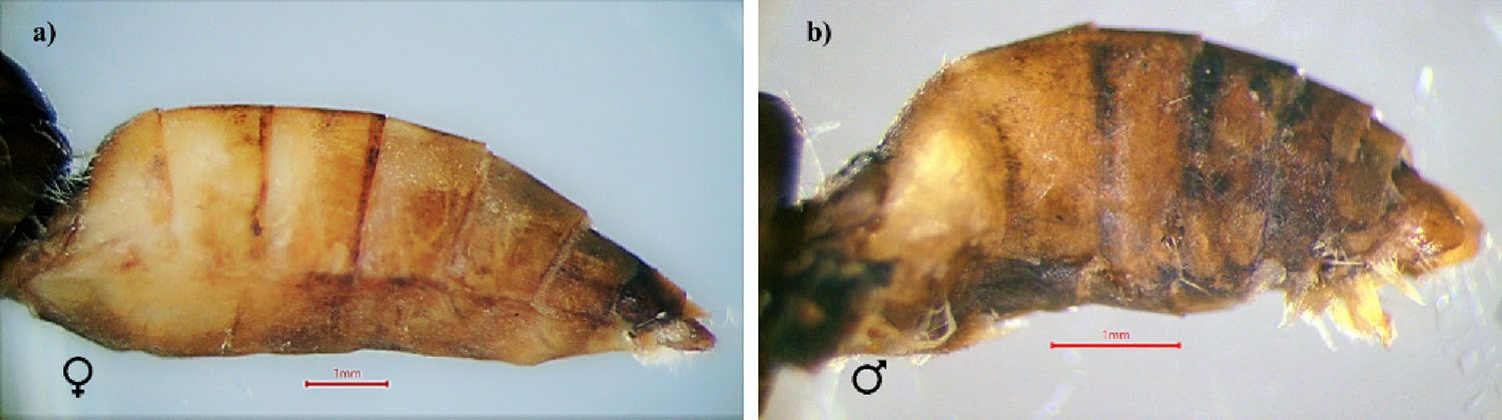
Abdomen description of pomegranate butterfly removed scales: (**a**) female and (**b**) male.

The number of abdominal segments is 10segments in both sexes. The last 3segments are greatly modified to form external genitalia. The female abdomen ends with short hairs (Fig. 11a). There is a dense tuft of long hairs (pencil hairs) at the end of the male abdomen associated with the scent gland and used for courtship (Fig. 11b).

#### Morphometrics of pomegranate butterfly

##### Body length (BL) measurements

The (BL) in both sexes was measured for each sex in the pomegranate butterfly (Table 1). In males, (BL) ranged from 11.360 to 12.000 mm with an average of 11.674 ± 0.299 mm, and in females ranged from 10.380 to 13.090 mm with an average of 11.458 ± 1.001 mm.

**Table 1:** Body length measurements from the head to the tip of the abdomen of the pomegranate butterfly.

Criteria (N = 5)	Body length measurements (mm)			
	Min	Max	Mean	± SD
Male	11.360	12.000	11.674	0.299
Female	10.380	13.090	11.458	1.001

##### Antennal length

The distance from the base to the tip of the antenna was measured for antennal length. The antennal length measurement of the pomegranate butterfly ranged from 6.190 to 7.850 mm with an average of 7.203 ± 0.529 mm. The above-mentioned measurements are based on ten individuals (n = 10).

##### Wings measurements

The (FWL) measurements ranged between 12.740–14.540 and 12.600–16.750 mm with an average of 13.728 ± 0.472 and 14.780 ± 1.164 mm for males and females, respectively.

The (HWL) measurements ranged between 7.820–9.940 and 8.990–12.370 mm with an average of 8.919 ± 0.716 and 10.446 ± 1.069 mm for males and females, respectively **(**Table 2**)**. The above measurements were based on ten individuals of each collection (n = 10) for both wings’ measurements.

**Table 2:** Wings length measurements fore wing length from humeral angle to apical angle and hind wing length from base to middle of apical margin in males and females for pomegranate butterfly.

Criteria (N = 10)		Wings length measurement (mm)			
		Min	Max	Mean	± SD
FW	♂	12.740	14.540	13.728	0.472
	♀	12.600	16.750	14.780	1.164
HW	♂	7.820	9.940	8.919	0.716
	♀	8.990	12.370	10.446	1,069

### Molecular identification of pomegranate butterfly

The first-time record of *Deudorix livia*, in the GenBank for the partial nucleotide sequence of *CO1* was deposited under the accession number MW463927. The sequencing of the mitochondrial *CO1* gene produced a nucleotide length of 632 bp. The nucleotide frequencies of adenine (A), thymine (T), cytosine (C), and guanine (G) were 33.7, 37.8, 14.8, and 13.7%, respectively. The average A + T content was 71.5%, which was higher than the average C + G content **(**Table 3**)**.

**Table 3:** Accession numbers, nucleotide frequencies, A + T and C + G content of mitochondrial *CO1* gene of *Deudorix livia*.

Specimens	Accession number	Base pair length	Nucleotide (%)				A + T content (%)	C + G content (%)
			A	T	C	G		
DAUF-Pomegranate	MW463927	632	33.7	37.8	14.8	13.7	71.5	28.5

The sequences of *CO1* of *D*. *livia* were subjected to BLAST/N at (NCBI) and revealed eight related species from the genus *Deudorix*; in addition to the out-group species; S*abatinca demissa* (HM431779.1), *Sabatinca calliarcha* (HM431781.1) and Sa*batinca heighwayi* (HQ575067.1) of the family Micropterigidae **(**Table 4**)**.

**Table 4:** The understudied *Deudorix livia* with their related species from the GenBank/NCBI in addition to the out-group species based on (*COX1*) sequences.

No	Species	Accession number
1	*Deudorix livia* isolate DAUF-Pomegranate cytochrome c oxidase subunit I (*COX1*) gene partial cds mitochondrial	MW463927
2	*Deudorix isocrates* isolate Tmk cytochrome oxidase subunit I (*COI*) gene partial cds mitochondrial	KX008608
3	*Deudorix diovis* voucher USNM ENT 00666518 cytochrome oxidase subunit 1 (*COI*) gene partial cds mitochondrial	GU695456
4	*Deudorix epijarbas* voucher 11ANIC-06219 cytochrome oxidase subunit 1 (*COI*) gene partial cds mitochondrial	JN286135
5	*Deudorix staudingeri* voucher UMKL-JJW0435 cytochrome oxidase subunit 1 (*COI*) gene partial cds mitochondrial	KF226398
6	*Deudorix littoralis* voucher USNM ENT 00666527 cytochrome oxidase subunit 1 (*COI*) gene partial cds mitochondrial	GU695463
7	*Deudorix smilis* voucher 11ANIC-06228 cytochrome oxidase subunit 1 (*COI*) gene partial cds mitochondrial	KF394006
8	*Deudorix democles* voucher 11ANIC-06225 cytochrome oxidase subunit 1 (*COI*) gene partial cds mitochondrial	JN286138
9	*Deudorix epirus* voucher USNM ENT 0066804 cytochrome oxidase subunit 1 (*COI*) gene partial cds mitochondrial	GU695459
10	*Sabatinca demissa* voucher CCDB-02223-D01 cytochrome oxidase subunit 1 (*COI*) gene partial cds mitochondrial	HM431779.1
11	*Sabatinca calliarcha* voucher CCDB-02223-D04 cytochrome oxidase subunit 1 (*COI*) gene partial cds mitochondrial	HM431781.1
12	*Sabatinca heighwayi* voucher CCDB-08380-G05 cytochrome oxidase subunit 1 (*COI*) gene partial cds mitochondrial	HQ575067.1

Among the studied *D*. *livia* species pairwise genetic distances ranged from 0.0114 to 0.0151. The most related species to our sample was *Deudorix isocrates*, where the genetic distance was 0.0114. The most genetic distance to our sample was *Deudorix epirus*, where the genetic distance was 0.0141. Overall, the mean distance value was 0.17% (Table 5).

**Table 5:** Pairwise distances using the (*COX1*) gene among *Deudorix livia* with their related species, and the out-group.

	1	2	3	4	5	6	7	8	9	10	11	12
MW463927.1_*Deudorix_livia*		0.0114	0.0126	0.0122	0.0122	0.0131	0.0130	0.0126	0.0141	0.0349	0.0352	0.0336
KX008608.1_*Deudorix_isocrates*	0.0678		0.0116	0.0118	0.0116	0.0122	0.0115	0.0108	0.0147	0.0353	0.0375	0.0364
GU695456.1_*Deudorix_diovis*	0.0739	0.0659		0.0068	0.0069	0.0072	0.0127	0.0122	0.0121	0.0342	0.0357	0.0334
JN286135.1_*Deudorix_epijarbas*	0.0739	0.0659	0.0263		0.0068	0.0074	0.0119	0.0119	0.0115	0.0345	0.0347	0.0331
KF226398.1_*Deudorix_staudingeri*	0.0719	0.0680	0.0263	0.0245		0.0072	0.0124	0.0118	0.0113	0.0329	0.0343	0.0320
GU695463.1*_Deudorix_littoralis*	0.0760	0.0700	0.0263	0.0281	0.0263		0.0119	0.0114	0.0121	0.0335	0.0348	0.0330
KF394006.1_*Deudorix_smilis*	0.0766	0.0625	0.0722	0.0640	0.0702	0.0661		0.0074	0.0151	0.0342	0.0346	0.0315
JN286138.1_*Deudorix_democles*	0.0764	0.0584	0.0703	0.0661	0.0683	0.0642	0.0282		0.0142	0.0337	0.0340	0.0315
GU695466.1_*Deudorix_epirus*	0.0932	0.0996	0.0703	0.0642	0.0622	0.0700	0.1002	0.0910		0.0379	0.0381	0.0356
HM431779.1*_Sabatinca_demissa*	0.3119	0.3188	0.3019	0.3052	0.2887	0.2953	0.3087	0.2987	0.3368		0.0198	0.0187
HM431781.1_*Sabatinca_calliarcha*	0.3153	0.3268	0.3188	0.3050	0.3086	0.3052	0.3094	0.2960	0.3405	0.1591		0.0140
HQ575067.1_*Sabatinca_heighwayi*	0.2953	0.3190	0.2984	0.2918	0.2822	0.2886	0.2828	0.2733	0.3229	0.1396	0.0851	

**Figure 12:**
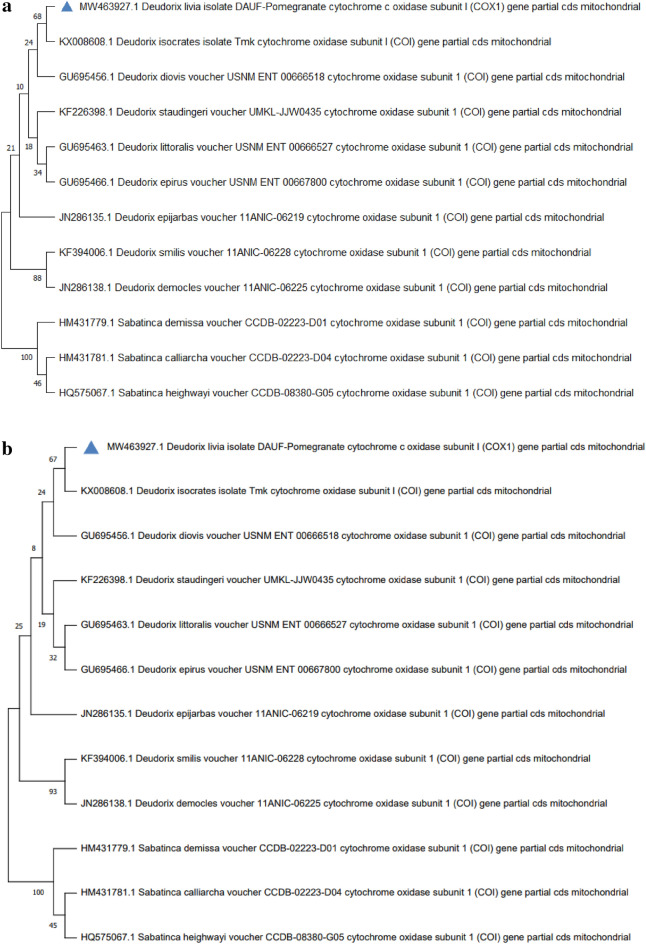
(**a**) Minimum-evolution phylogenetic tree among *Deudorix livia* with their related species, and the out-group using the (*COX1*) gene. (**b**) Neighbour Joining phylogenetic tree among *Deudorix livia* with their related species, and the out-group using (*COX1*) gene.

To conduct the phylogenetic tree analysis using *CO1* sequencing, *D*. *livia* was submitted to be analyzed together with eight related *Deudorix* species sequences and the out-group species from GenBank/NCBI (previously mentioned in Table 5). For more illustrative phylogenetic relations, more than one phylogenetic method was used (ME and NJ) based on the *CO1* gene. These methods showed nearly the same relations with some differences in support values and revealed 3 main features: (1) species of out-group formed a separate cluster. (2) all *Deudorix* species formed two main clades; the first includes *D*. s*milis* and *D*. *democles* while the second contains the rest species. (3) the most related species to *D*. *livia* crops was *D*. *isocrates* (Fig. 12a,b).

## Discussions

The Lycaenidae is a family comprising several thousand species. Many are brightly colored, and this is often the result of interference effects caused by the microstructure of the wings. One of the genera in this family is the *Deudorix* genus. This genus is formed by many species complexes that can be difficult to identify due to the similar shapes of species in this genus. *Deudorix livia* was described by Klug in 1834 based on the *Lycaena* genus, then the *Virachola* genus by Moore in 1881. Several studies about this genus were concerned ecology, economic harm, and control^1,8,18–21^, but studies on the morphology of this pest are rare. Also, morphological studies were superficial, such as^22–24^. So, the current study aims to accurately identify the pomegranate butterfly type through a full description of morphological and genetic techniques.

Although morphometric identification is much better than general morphology of insect’s species, using genetic tools became the most accurate method to differentiate between closely related species. Our results indicated that *Deudorix livia* was recorded for the first time in the GenBank for the partial nucleotide sequences of *CO1* deposited under accession number MW463927. However, among the studied *D. livia* species, pairwise genetic distances ranged from 0.0114 to 0.0151. The most related species to our sample was *Deudorix isocrates*, although it differed morphologically from our sample^25^, where the genetic distance was 0.0114. Based on the finding of Kaleshkumar^26^ who reported that closely related species have the lowest genetic distance, while the highest genetic distance refers to highly diverged cases.

Some morphological features were studied for the first time, such as compound eyes focused on one scanned eye and interfacial hairs found between every three ommatidia. Meanwhile, most researchers focused on the general description of the eye as having dense white and dark scales on the borders, and others mentioned that it doesn’t have ocelli^9,17,27^. Also, on the upper side of the male hindwing, there was one spotted patch in the sub-discal area and near the humeral angle (basal area). This feature is believed to be the female sexual pheromone receiving area during the mating process. So, it needs extensive future studies to identify such pheromones for using alternative pesticides to help control this pest.

The importance of real nomenclature for the pest is one of the most important steps for conducting appropriate control approach to reduce the population density of pomegranate butterfly below the economic injury level.

## Conclusions

The findings indicate that both sexes' body scales are white on the ventral side and a mixture of dark and light grey on the dorsal side. Also, it contributes a similar wing venation. However, the average body length (BL) for male was 11.674 0.299 mm, while for female it was 11.458 1.001 mm. The mitochondrial *CO1* gene from *Deudorix livia* was originally partially sequenced and deposited in GenBank under the accession number MW463927. The accurate identification of *D*. *livia* in pomegranate agroecosystems can help in designing appropriate (IPM) programs for this serious economic pest. This is in line with the global goals of sustainable development for environmental integrity and human health.

## Data Availability

The datasets analyzed during the current study are available in the [GenBank/NCBI] repository, WEB LINK [https://www.ncbi.nlm.nih.gov/nuccore/MW463927.1/], ACCESSION NUMBER [MW463927.1].

## Abbreviations

IPM: Integrated pest management
Sc: Subcostal
R: Radius
M: Media
Cu: Cubitus
A: Anal
FW: Forewing
HW: Hindwing
BL: Body length
FWL: Forewing length
HWL: Hindwing length
AntL: Antennal length
*CO1*: Cytochrome c oxidase subunit 1
A: Adenine
C: Cytosine
T: Thymine
G: Guanine
NCBI: National Center for Biotechnology Information
NJ: Neighbour-joining
ME: Minimum evolution
